# H-NS controls the susceptibility of *Escherichia coli* to aminoglycosides by interfering its uptake and efflux

**DOI:** 10.3389/fvets.2025.1534498

**Published:** 2025-02-06

**Authors:** Qiuru Chen, Yulei Liang, Yanbin Dong, Junling Cui, Kun He, Xiaoyuan Ma, Jinfeng Zhao, Yajun Zhai, Li Yuan

**Affiliations:** ^1^College of Veterinary Medicine, Henan Agricultural University, Zhengzhou, China; ^2^Key Laboratory of Quality and Safety Control of Poultry Products (Zhengzhou), Ministry of Agriculture and Rural Affairs, Zhengzhou, China; ^3^Ministry of Education, Key Laboratory for Animal Pathogens and Biosafety, Zhengzhou, China; ^4^Zhengzhou Key Laboratory of Research and Evaluation of Traditional Chinese Veterinary Medicine, Zhengzhou, China

**Keywords:** H-NS, aminoglycosides, uptake, efflux, glycolysis, proton motive force

## Abstract

H-NS is a histone-like nucleoid-structuring protein that regulates gene expressions, particularly acquired foreign genes, however, little is known about whether H-NS can modulate bacterial susceptibility by regulating its intrinsic genes. The *hns*-deleted mutant EΔ*hns*, the *hns*-complemented strain EΔ*hns*/p*hns* and the *hns*-overexpressed strain E/p*hns* were derivatives of *Escherichia coli* ATCC 25922, the susceptibility of which were assessed by the broth microdilution method and time-kill curves assays. We found that the MICs for strain EΔ*hns* against gentamicin and amikacin were significantly decreased by 8–16 folds in contrast to *E. coli* ATCC 25922. Further studies displayed that the absence of *hns* caused damage to the bacterial outer membrane and increased the expression levels of porin-related genes, such as *ompC, ompF, ompG*, and *ompN*, thus obviously enhancing aminoglycosides uptake of strain EΔ*hns*. Meanwhile, *hns* deletion also led to remarkable inhibition of the efflux pumps activity and decreased expressions of efflux-related genes *clbM, acrA, acrB, acrD*, and *emrE*, which reduced the efflux of aminoglycosides. In addition, the activation of glycolysis and electron transport chain, as well as the reduction of Δψ dissipation, could lead to a remarkable increase in proton motive force (PMF), thus further inducing more aminoglycosides uptake by strain EΔ*hns*. Our findings reveal that H-NS regulates the resistance of *E. coli* to aminoglycosides by influencing its uptake and efflux, which will enrich our understanding of the mechanism by which H-NS modulates bacterial resistance.

## 1 Introduction

The histone-like nucleoid structuring (H-NS) protein is 137 amino acids in length in *Escherichia coli* and closely related bacteria and is present at very high levels, with approximately 20,000 to 60,000 molecules per cell (1, 2). It is generally believed that the H-NS protein, as a global regulator, preferentially binds to AT-rich DNA by preventing RNA polymerase from accessing or escaping promoters (3), thereby silencing the expression of corresponding genes, notably acquired foreign genes, such as resistance genes acquired by horizontal transfer (4, 5). To date, H-NS has received considerable attention in regulating acquired foreign genes, and it has been proved that H-NS protects bacteria and drives their evolution via regulating the expression of foreign DNA (6–8). Meanwhile, some literatures have clarified that H-NS can also modulate genes associated with virulence, stress response, quorum sensing, biosynthesis pathways and cell adhesion by recognizing intrinsically curved DNA (9–11).

Up to now, there are a few reports on H-NS regulating multidrug resistance by controlling the inherent genes in the bacterial host. Nishino et al. proposed that H-NS contributed to multidrug resistance *E. coli* by regulating the expression of multidrug exporter genes such as *acrEF* and *mdtEF* (12, 13). In 2018, Deveson Lucas team found that H-NS inactivation resulted in an increased resistance to colistin in a clinical isolate of *Acinetobacter baumannii* (14). Rodgers et al. proved that H-NS modulated antibiotic resistance in *Acinetobacter baumannii* by governing genes codifying for biofilms and efflux pumps (15). Antibiotic resistance is achieved through a variety of mechanisms, such as alteration or bypass of the drug target, production of antibiotic-modifying enzymes, decreased drug uptake, increased drug efflux, and biofilm formation (16). However, little is known about whether H-NS can influence multidrug resistance by interfering drug uptakes.

Aminoglycosides have been one of the important antibiotics for preventing Gram-negative bacteria infection since 1940. Nevertheless, the widespread presence of resistance bacteria has led to a sharp decline in its efficacy. Recently, we occasionally found that the deletion of *hns* increased the susceptibility of *E. coli* to many antibiotics, especially aminoglycosides, with their minimal inhibitory concentrations (MICs) decreased by 8–16 folds. The work described below elucidates that H-NS regulates the resistance of *E. coli* ATCC 25922 to aminoglycosides by influencing its uptake and efflux, which will enrich our understanding of the regulatory mechanism of H-NS on bacterial resistance, and also contribute to the development of new drugs to curb bacterial resistance.

## 2 Materials and methods

### 2.1 Bacterial strains, plasmids, and primers

Bacteria and plasmids used in this study are listed in Table 1. *Escherichia coli* ATCC 25922 was obtained from the China Institute of Veterinary Drug Control. Strain EΔ*hns* is a derivative of *E. coli* ATCC 25922 via the one-step inactivation of chromosomal gene *hns* (17). The complementary strain EΔ*hns*/p*hns* was constructed as follows: Firstly, the complete open reading frame of *hns* was amplified by PCR from the genomic DNA of *E. coli* ATCC 25922 using primers *Xho*I-*hns*–F/*Hind*III-*hns*-R (Table 2). Thereafter, the expression plasmid pBAD::*hns* was generated by inserting the target fragment to the vector pBAD (Karsbad, CA, the United States) and then was introduced to EΔ*hns* by electroporation. The overexpressed *hns* strain E/p*hns* is a derivative of *E. coli* ATCC 25922 that was introduced of pBAD::*hns* by electroporation. All strains were cultured in fresh Luria-Bertani (LB) broth (Beijing Land Bridge Technology Co., Ltd.) at 37°C, where strains EΔ*hns*, EΔ*hns*/p*hns* and E/p*hns* were induced by 0.2% L-arabinose (18).

**Table 1:** The MICs of antimicrobial agents against the tested strains (μg/mL).

**Antimicrobial agents**	***E. coli ATCC 25922***	***EΔ*hns**	***EΔ*hns/phns**	***E/p*hns**
Gentamicin	1.000	0.063	0.125	1.000
Amikacin	1.000	0.125	0.250	1.000
Doxycycline	0.500	0.250	0.500	0.500
Tigecycline	0.063	0.031	0.063	0.063
Florfenicol	2.000	1.000	2.000	2.000
Cefotaxime	0.031	0.016	0.016	0.031
Enrofloxacin	0.008	0.002	0.008	0.008

Strain EΔ*hns* is the derivative of *E. coli* ATCC 25922 that lacks *hns*, strain EΔ*hns*/p*hns* is the derivative of EΔ*hns* that carries the recombined plasmid pBAD::*hns*, while strain E/p*hns* is the derivative of *E. coli* ATCC 25922 that carries pBAD::*hns*. Strains EΔ*hns*, EΔ*hns*/p*hns* and E/p*hns* are induced by 0.2% L-arabinose.

**Table 2:** The strains, plasmids and primers used in this study.

**Strains/plasmids/primers**	**Relevant characteristics**	**References/length (bp)**
**Strains**		
*E. coli* ATCC 25922	Supplied by China Institute of Veterinary Drug Control	
EΔ*hns*	Single deletion strain, derivatives of *E. coli* ATCC 25922 that lacks *hns*	(17)
pBAD::*hns*	*hns* is cloned to pBAD/HisA; Amp^r^	This study
EΔ*hns*/p*hns*	Derivative of EΔ*hns* that carries pBAD::*hns*.	This study
E/p*hns*	Derivative of *E. coli* ATCC 25922 that carries pBAD::*hns*.	This study
**Plasmids**		
pKD4	Vector for lambda red-mediated mutagenesis; Kan^r^	(18)
pKD46	Vectorfor lambda red-mediated mutagenesis, λ-red expression from arabinose-inducible promoter; temperature sensitive; Amp^r^	(18)
pCP20	Vector for lambda red-mediated mutagenesis; Amp^r^	(18)
pBAD/HisA	Expression vector; Amp^r^	(18)
**Primers for amplifying the** ***hns*** **complete open reading frame**		
*Xho* I-*hns*-F	ATT*CTCGAG*ATGAGCGAAGCACTTAAA	432
*Hind* III- *hns*-R	GCG*AAGCTT*TTATTGCTTGATCAGGAA	
**Real-time relative quantitative PCR primers**		
*hns-*F	GACGGTATTGACCCGAACGA	119
*hns-*R	TTAGTTTCGCCGTTTTCGTC	
*ompC*-F	CTACAGACGGACGCAGACCAA	113
*ompC*-R	CACCCAGACCTACAACGCAACT	
o*mpF*-F	GTTGGCGGCGTTGCTACCTATC	194
*ompF*-R	GTACGGTCAGCGGCACCATAAG	
*ompG*-F	GCTGGATCGCTGGAGTAACTGG	160
*ompG*-R	GCTGTCGCCTTCGTCGTGAT	
*ompN*-F	AGGTAACAACGAAGGTGCCAGT	152
*ompN*-R	TGCGGTCAGAAGAGGTGTATGC	
*acrA*-F	GGCGATAAGTGGCTGGTGACA	135
*acrA*-R	GCTTGCGGCTTGCTGGTTAT	
*acrB*-F	CGAGAAGAGCACGCACCACTAC	120
*acrB*-R	GGCAGACGCACGAACAGATAGG	
*clbM-F*	GTATCATGGCACTGGCACTACC	103
*clbM-R*	ATCAGCGTCAACAACACCGAAT	
*tolC-F*	CAGCAAGCACGCCTTAGTAACC	169
*tolC-R*	CGTTAGAGTTGATGCCGTTCGC	
*emrE-F*	TCTGGTCAGGAGTCGGTATCGT	86
*emrE-R*	GCCTATGATAGCTGGCAGGTCC	
*ompW-F*	AGGTGCTGGTGGTACGTTAGGA	193
*ompW-R*	CAGTGTTGGCGGCAGATGATGA	
*16S-F*	CCTCAGCACATTGACGTTAC	(18)
*16S-R*	TTCCTCCAGATCTCTACGCA	

The underlined bases are restriction sites. Kan^r^ and Amp^r^ indicate resistance to kanamycin and ampicillin. Strains EΔ*hns*, pBAD::*hns*, EΔ*hns*/p*hns* and E/p*hns* are induced by 0.2% L-arabinose.

### 2.2 Real-time relative quantitative PCR analysis

A single colony of strains, such as *E. coli* ATCC 25922, EΔ*hns*, EΔ*hns*/p*hns* and E/p*hns* were cultured in LB medium at 37°C. After growth overnight, the cultures were diluted 1:100 in fresh medium and grown to an OD_600_ of 0.5. Following this, the total bacterial RNA was extracted with a TaKaRa MiniBEST universal RNA extraction kit (TaKaRa Bio, Inc., Shiga, Japan). The quantity of extracted RNA was determined by A_260_ measurements and purity was evaluated by the A_260_/A_280_ ratio using a NanoDrop 1000 Spectrophotometer (Thermo Scientific, Hvidovre, Denmark). The cDNA samples were synthesized using the cDNA reverse transcription kit with gDNA Eraser (TaKaRa Bio, Inc.) and then the RT-qPCR was performed with an annealing temperature of 60°C according to our previous study (18). The relative expression levels were calculated by the 2^−ΔΔCt^ method and compared with the levels of *E. coli* ATCC 25922. The *16S rRNA* gene was chosen as a housekeeping gene, and the primers of RT-qPCR are listed in Table 1. Three independent biological replicates were performed.

### 2.3 Growth curve assay

Growth curves for *E*. *coli* ATCC 25922, EΔ*hns*, EΔ*hns*/p*hns* and E/p*hns* were plotted. Overnight cultures were inoculated in fresh LB medium and grown to an OD_600_ of 0.5. Then the cultures were diluted (1:1,000) in preheated fresh LB broth and inoculated with shaking at 37°C. The OD_600_ was measured periodically at 1-h intervals using an ultraviolet spectrophotometer and shown as means ± standard deviations. Experiments were performed with at least three biological replicates.

### 2.4 Antimicrobial susceptibility testing

Antimicrobial susceptibility testing was tested using the broth microdilution method according to the guidelines of Clinical and Laboratory Standards Institute (CLSI) guidelines (19). The antimicrobial agents are cefotaxime, gentamicin, amikacin, doxycycline, tigecycline, florfenicol, and enrofloxacin. Experiments were performed with at least three biological replicates.

### 2.5 Time-dependent killing assay *in vitro*

Overnight cultures of *E*. *coli* ATCC 25922, EΔ*hns* and EΔ*hns*/p*hns* were diluted (1:1,000) in fresh LB broth and grown to an OD_600_ of 0.5. Bacteria were cultured in the LB broth with different concentrations of gentamicin (0.25, 0.5, 1, and 2 μg/mL) for 24 h. At regular intervals, the culture broths were serially diluted with 0.9% saline and plated onto LB agar plates. Colony forming units (CFU) were counted after 18 h of incubation at 37°C, and the Log_10_ CFU values were calculated according to previous study (20). Take the Log_10_ CFU values as the vertical axis, and the incubation time (h) as the horizontal axis to establish the time sterilization curve. Three independent biological replicates were performed.

### 2.6 Outer membrane permeability assay

Overnight cultures of *E*. *coli* ATCC 25922, EΔ*hns* and EΔ*hns*/p*hns* were diluted 1:100 in fresh LB medium and incubated at 37°C. Samples equivalent to an OD_600_ of 0.5 were removed and suspended with 5 mmol·L^−1^ N-2-hydroxyethylpiperazine-N'-2- ethanesulfonic acid (HEPES, pH 7.0, plus 5 mmol·L^−1^glucose). Thereafter, the OD_600_ of the above bacteria suspension was standardized to 0.5 using the same buffer. Fluorescent probe NPN (10 μM) was used to evaluate the OM integrity of strains (21). Fluorescence intensity was measured using a Sparpk 10M Microplate reader (Tecan) with the excitation wavelength at 350 nm and the emission wavelength at 420 nm.

### 2.7 Scanning electron microscopy analysis

The culture suspension of *E. coli* ATCC 25922 and EΔ*hns* were prepared according to the method described in 2.6. Thereafter, the bacterial suspensions were centrifuged, washed softly with phosphate-buffered saline (PBS) buffer for three times, and mixed with 2.5% (w/v) glutaraldehyde in 0.1 M PBS buffer at 25°C for about 2 h away from light. Subsequently, the samples were dehydrated in sequential with a graded ethanol series (30%, 50%, 70%, 90%, and 100%), transferred to a culture dish, dried overnight, and observed using SEM (Hitachi, Japan).

### 2.8 Bacterial membrane potential determination

Bacteria were cultured and standardized to an OD_600_ of 0.5 using similar protocols described in 2.6. DiSC_3_ (5) (0.5 μM) (Aladdin, Shanghai, China) was added to achieve a final concentration of 2 μM in the mixture to determine the transmembrane electric potential (Δψ). The dissipation for Δψ in *E*. *coli* ATCC 25922, EΔ*hns* and EΔ*hns*/p*hns* was measured with the excitation wavelength of 622 nm and emission wavelength of 670 nm according to the method described in the previous study (22).

### 2.9 Efflux pumps assay

The accumulation of ethidium bromide (Beyotime, Shanghai, China) in the cells was monitored as previously described with some modifications (21). Strains of *E*. *coli* ATCC 25922, EΔ*hns* and EΔ*hns*/p*hns* were grown to an OD_600_ of 0.5, then centrifuged and suspended with 5 mmol·L^−1^ HEPES (pH 7.0, plus 5 mmol·L^−1^glucose). Subsequently, the OD_600_ of the bacterial suspension was standardized to 0.3 using the same buffer. 5 μM of ethidium bromide was added to determine the efflux pumps activity. Fluorescence intensity was determined with a Spark multifunctional microplate reader with excitation wavelength 530 nm and emission wavelength 600 nm. Each experiment was conducted in triplicate at least three times.

### 2.10 Transcriptomic analyses

Total RNA of strains *E*. *coli* ATCC 25922 and EΔ*hns* were extracted by the RNAprep Pure Cell/Bacteria Kit (TIANGEN Biotech (Beijing) Co., Ltd) and sequenced using the Illumina NovaSeq 2000 system by Novogene Bioinformatics Technology Co., Ltd. Differentially expressed genes (DEGs) were identified by using the fragments per kilobase of transcript per million mapped reads method with *P*_adj_ < 0.05 and fold change (FC) of | log_2_FC | > 1. RNA-sequencing reads were aligned to *E. coli* ATCC 25922 (Ref-Seq accession no. CP025268).

### 2.11 Statistical analyses

Statistical analysis and the generation of plots were performed using GraphPad Prism 8. Unpaired *t*-test (normally distributed data) between two groups was used to calculate *P*-values. Differences with *P* < 0.05 were considered as significant difference. Significance levels are indicated by numbers of asterisks (ns, No significant difference, ^*^ *P* < 0.05, ^**^ *P* < 0.01, ^***^ *P* < 0.001, ^****^ *P* < 0.0001).

## 3 Results and discussion

### 3.1 The expression levels of *hns* gene in recombinant strains

**Figure 1:**
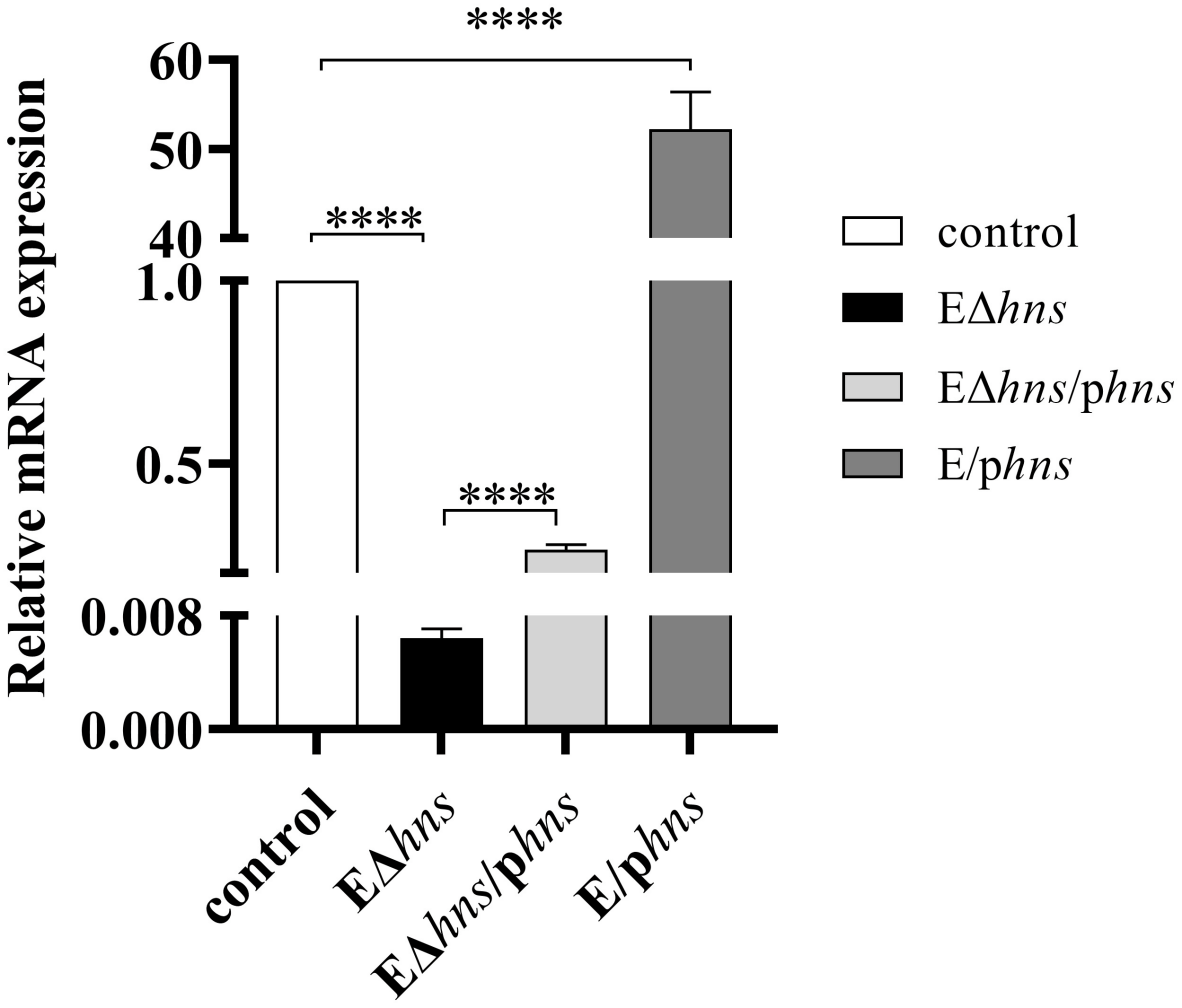
The mRNA expression levels of *hns* in the strains *E. coli* ATCC 25922, EΔ*hns*, EΔ*hns*/p*hns* and E/p*hns*. *E. coli* ATCC 25922 is the control. The strain EΔ*hns* is the derivative of *E. coli* ATCC 25922 that lacks *hns*, the strain EΔ*hns*/p*hns* is the derivative of EΔ*hns* that carries the recombined plasmid pBAD::*hns*, while the strain E/p*hns* is the derivative of *E. coli* ATCC 25922 that carries pBAD::*hns*. Strains EΔ*hns*, EΔ*hns*/p*hns* and E/p*hns* are induced by 0.2% L-arabinose. Data are expressed as the mean ± standard deviation (SD) (**** *P* < 0.0001).

The mRNA expression levels of *hns* gene in *E*. *coli* ATCC 25922, *hns* deletion strain EΔ*hns*, complemented strain EΔ*hns*/p*hns* and overexpressed strain E/p*hns* were measured by RT-qPCR (Figure 1). Compared with strain EΔ*hns*, the *hns* expressions of strain EΔ*hns*/p*hns* increased by 44-fold, from 0.006 ± 0.001 increasing to 0.264 ± 0.015, although they were still sharply lower than that of *E. coli* ATCC 25922 (*P* < 0.0001), demonstrating that the complement of *hns* could only partially restore the function of H-NS in the deletion strain EΔ*hns*, which may be closely linked to the location of H-NS, such as on chromosome or plasmids (1, 2, 23). Meanwhile, we observed that the *hns* expressions of strain E/p*hns* (52.232 ± 4.182) were 52.23-fold increments in comparison with the reference strain *E. coli* ATCC 25922, proving that strain E/p*hns* overexpressed *hns*.

### 3.2 The deletion of *hns* has obviously impaired the bacterial adaptability

**Figure 2:**
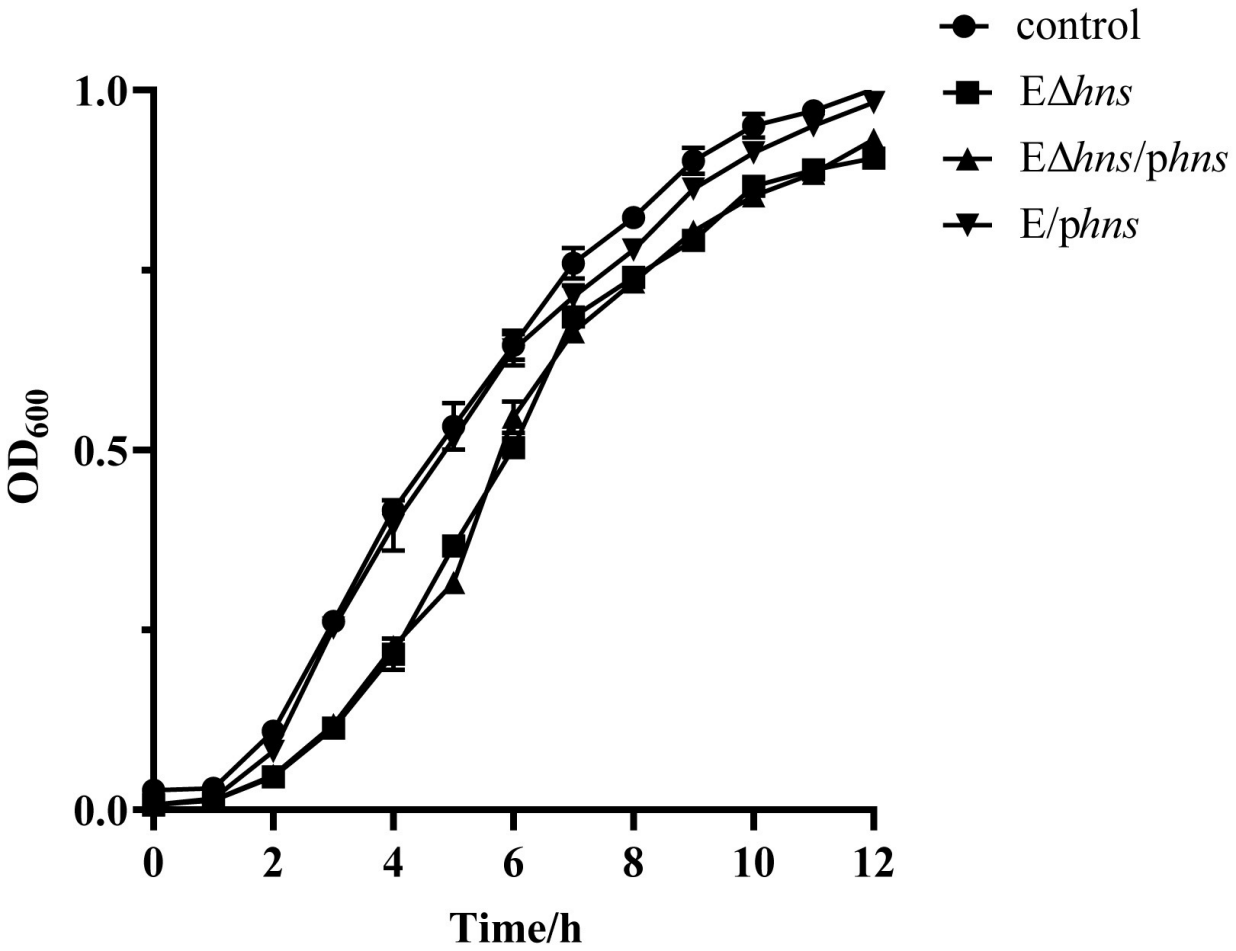
Growth curves of the strains *E. coli* ATCC 25922, EΔ*hns*, EΔ*hns*/p*hns* and E/p*hns. E. coli* ATCC 25922 is the control. The strain EΔ*hns* is the derivative of *E. coli* ATCC 25922 that lacks *hns*, the strain EΔ*hns*/p*hns* is the derivative of EΔ*hns* that carries the recombined plasmid pBAD::*hns*, while the strain E/p*hns* is the derivative of *E. coli* ATCC 25922 that carries pBAD::*hns*. Curves indicate the means of three independent experiments. Strains EΔ*hns*, EΔ*hns*/p*hns*, and E/p*hns* are induced by 0.2% L-arabinose.

Growth kinetics of strains *E*. *coli* ATCC 25922, EΔ*hns*, EΔ*hns*/p*hns* and E/p*hns* were established respectively based on optical density at 600 nm values (Figure 2). No difference in growth was observed between *E*. *coli* ATCC 25922 and E/p*hns*, which inferred that *hns* overexpressions had no effect on the growth of *E. coli*. It's worth noting that the OD_600_ values of strains EΔ*hns* and EΔ*hns*/p*hns* were significantly lower than those of *E*. *coli* ATCC 25922 at 3–6 h incubation time, although the OD_600_ values rose to the control level at the later incubation, in line with previous studies (24). The aforementioned results reflect that *hns* deletion will lead to reduced adaptability of *E. coli*, although it does not affect the bacterial survival.

### 3.3 The deletion of *hns* can increase the susceptibility of *E. coli*

To determine whether *hns* deletion alters the susceptibility of *E. coli* to a variety of drugs, such as gentamicin, amikacin, cefotaxime, doxycycline, tigecycline, florfenicol, and enrofloxacin, the MIC values were determined for *E. coli* ATCC 25922, EΔ*hns*, EΔ*hns*/p*hns* and E/p*hns* (Table 1). In contrast to *E. coli* ATCC 25922 (MICs = 0.008 ~ 2 μg/mL), the MICs for strain EΔ*hns* were significantly decreased, with gentamicin decreased by 16 folds, followed by amikacin (8-fold) and enrofloxacin (4-fold), while cefotaxime, doxycycline, tigecycline and florfenicol displaying 2-fold decreases. As expected, the MICs for strain EΔ*hns*/p*hns* partially recovered, while for strain E/p*hns* was the same as that of the control. Collectively, our data illustrate that protein H-NS negatively regulates the multidrug resistance of *E. coli*.

### 3.4 Gentamicin displays dramatically bactericidal effect on strain EΔ*hns*

**Figure 3:**
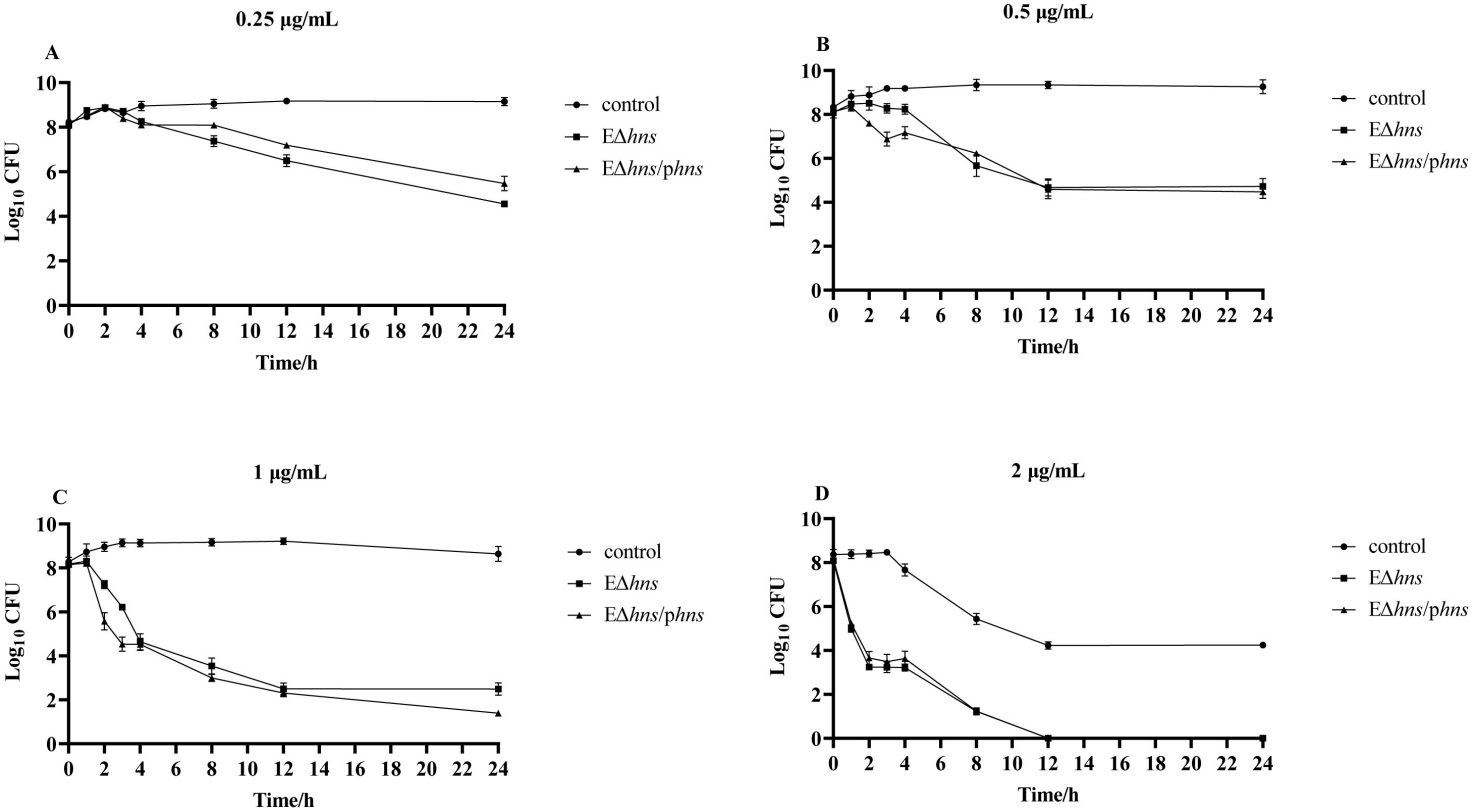
Time-kill curves for exponential phase strains *E. coli* ATCC 25922, EΔ*hns* and EΔ*hns*/p*hns*, in the presence of gentamicin. The values of Log_10_ CFU are plotted as a function of time for different concentrations of gentamicin: **(A)** 0.25, **(B)** 0.5, **(C)** 1, and **(D)** 2 μg/mL). *E. coli* ATCC 25922 is the control. The strain EΔ*hns* is the derivative of *E. coli* ATCC 25922 that lacks *hns* and the strain EΔ*hns*/p*hns* is the derivative of EΔ*hns* that carries the recombined plasmid pBAD:*hns*. Strains EΔ*hns* and EΔ*hns*/p*hns* are induced by 0.2% L-arabinose.

To further investigate the impact of *hns* deletion on the susceptibility of strains, we conducted *in vitro* time-dependent killing assay of gentamicin against *E. coli* ATCC 25922, EΔ*hns* and EΔ*hns*/p*hns*. As shown in Figure 3, strains *E. coli* ATCC 25922, EΔ*hns* and EΔ*hns*/p*hns* were inhibited in a concentration-dependent manner after incubation with different concentrations of gentamicin from 0 h to 24 h. In addition, the bactericidal effect of gentamicin against EΔ*hns* displayed markedly stronger than that against *E. coli* ATCC 25922. For strain EΔ*hns*, the number of viable bacteria began to decrease obviously after incubation with 0.25 μg/mL of gentamicin for 3 h (Figure 3A), and all died after incubation with 2 μg/mL for 12 h (Figures 3C, D), whereas for *E. coli* ATCC 25922, the bactericidal effect was observed only after incubation with 2 μg/mL of gentamicin for 4 h (Figure 3D). In conclusion, *hns* deletion leads to increased susceptibility of *E. coli* ATCC 25922 to gentamicin.

Notably, there was no significant difference in the bactericidal effect of gentamicin against strains EΔ*hns* and EΔ*hns*/p*hns*, suggesting that (1) the expressions of *hns* located on pBAD could not completely complement the deletion of *hns* on chromosome, which had been also confirmed by the expression of *hns* in recombinant bacteria (Figure 1) and the results of *in vitro* antimicrobial susceptibility testing (Table 1); (2) the deletion of *hns* gene might lead to bacterial damages that were difficult to reverse even after the gene *hns* was complemented, such as membrane damages, although further studies were needed.

### 3.5 Higher OM permeability contributes to drugs uptake of strain EΔ*hns*

**Figure 4:**
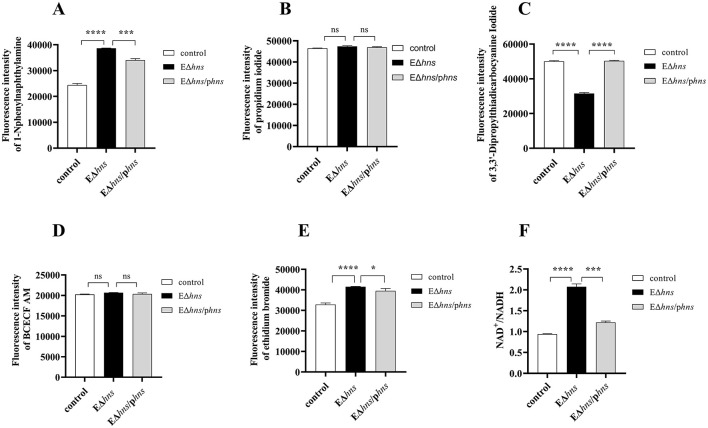
Effects of *hns* deleted from *E. coli* ATCC 25922 on bacterial membrane permeability, membrane potential (Δψ), efflux pumps activity and cellular respiration. **(A)** The permeabilities of outer membrane are evaluated by the fluorescence intensity with 1-N-phenylnaphthalamine. **(B)** The permeabilities of inner membrane are evaluated by the fluorescence intensity with propidium iodide. **(C)** The dissipations for Δψ are analyzed by the fluorescence intensity with 3,3′-Dipropylthiadicarbocyanine Iodide. **(D)** Membrane proton gradient is assessed by fluorescence dye BCECF-AM. **(E)** Efflux pumps activity is assessed by fluorescence dye ethidium bromide. **(F)** The changes of NAD^+^/NADH ratio are measured using an NAD^+^/NADH Assay Kit. *E. coli* ATCC 25922 is the control. The strain EΔ*hns* is the derivative of *E. coli* ATCC 25922 that lacks *hns* and the strain EΔ*hns*/p*hns* is the derivative of EΔ*hns* that carries the recombined plasmid pBAD::*hns*. Strains EΔ*hns* and EΔ*hns*/p*hns* are induced by 0.2% L-arabinose. All experiments are performed with biological replicates and present as the mean ± SD. The significances are determined by unpaired *t*-test between two groups. ns, No significant difference by Student's *t*-test; *, *P* < 0.05; **, *P* < 0.01; ***, *P* < 0.001; ****, *P* < 0.0001.

To understand the molecular mechanism by which H-NS regulates the susceptibility of *E. coli*, we determined the OM permeability changes in *E. coli* ATCC 25922, EΔ*hns* and EΔ*hns*/p*hns* using 1-N-phenylnaphthylamine (NPN) as a hydrophobic fluorescent probe (Figure 4A). The results demonstrated that strain EΔ*hns* exhibited dramatically stronger NPN uptakes than that of *E. coli* ATCC 25922, implying that the absence of *hns* led to the elevated OM permeability of *E. coli*. In parallel, compared with strain EΔ*hns*, the fluorescence values of complementary strain EΔ*hns*/p*hns* decreased significantly, although it still showed higher than *E. coli* ATCC 25922, which was in agreement with the expression levels of *hns* gene in the complementary strain. Soon afterwards, we further determined the permeability of inner membrane (IM) of the aforementioned bacteria using propidium iodide (PI) as a fluorescent probe (21) and no evident changes were observed (Figure 4B). In summary, *hns* deletion increases OM permeability of *E. coli*, however has little effect on IM.

### 3.6 Membrane damage improves the susceptibility of strain EΔ*hns*

**Figure 5:**
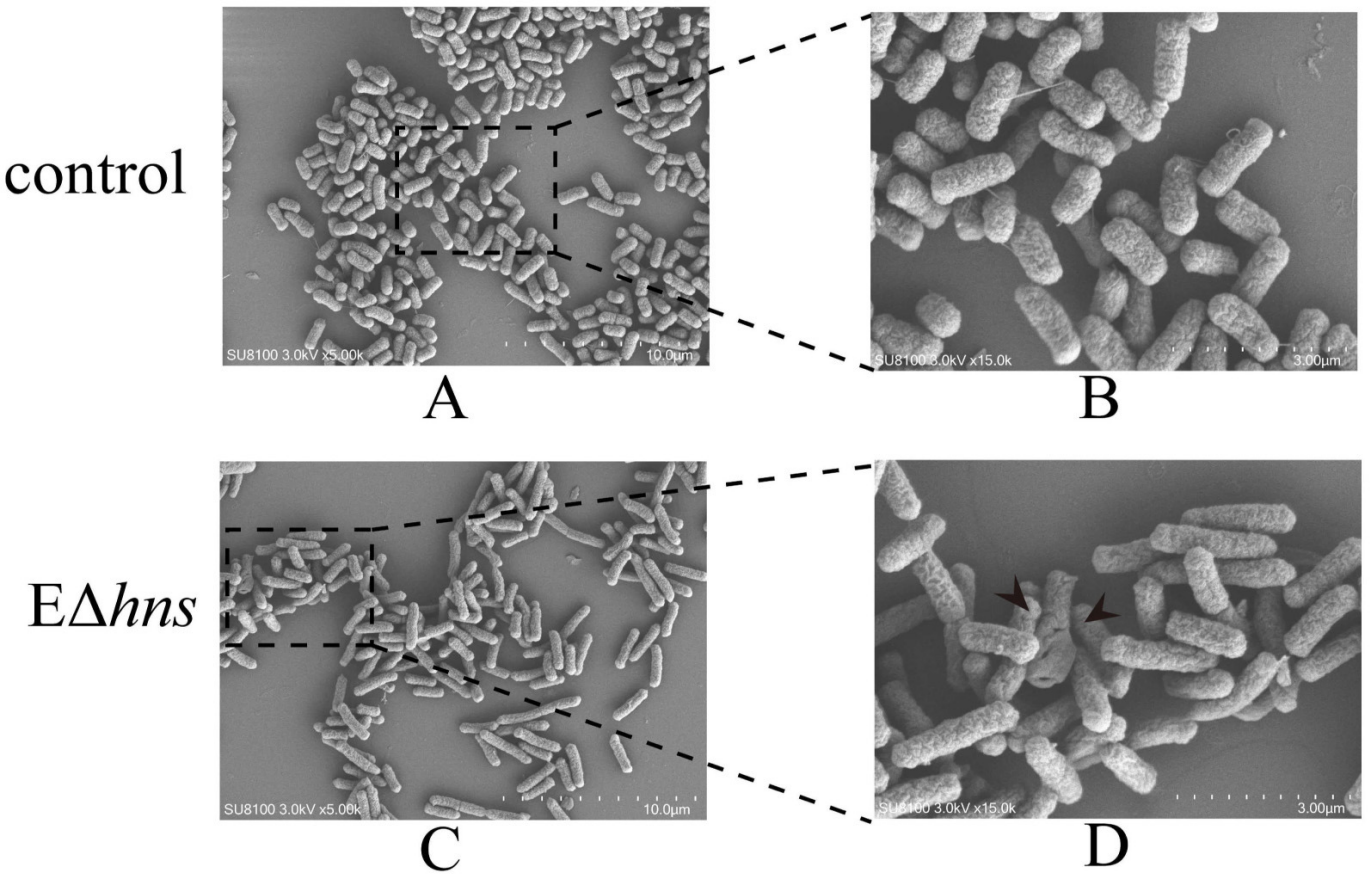
Morphological changes of the strains *E. coli* ATCC 25922 and EΔ*hns*. **(A, B)** Bacterial morphology of *E. coli* ATCC 25922; **(C, D)** bacterial morphology of EΔ*hns*. *E. coli* ATCC 25922 is the control. The strain EΔ*hns* is the derivative of *E. coli* ATCC 25922 that lacks *hns* and is induced by 0.2% L-arabinose.

To clarify the impact of *hns* deletion on the morphology of *E. coli*, morphological changes of strains *E. coli* ATCC 25922 and EΔ*hns* were examined by SEM. In comparison with *E. coli* ATCC 25922, the cell surface of strain EΔ*hns* exhibited different degree of deformations and devastation (Figure 5). As shown in Figure 5, long rod-shaped bacteria increased significantly in strain EΔ*hns*, speculating that it might be related to the slow growth caused by the deletion of the *hns* gene (Figures 5C, D). In addition, some cells of strain EΔ*hns* displayed observable membrane damage, which may be one of the reasons for the increased uptake of aminoglycosides by strain EΔ*hns*. Recently, Lv et al. proved that hypoionic shock-induced cell membrane damage could dramatically increase the bacterial uptake of aminoglycosides, which enhanced bactericidal action of the antibiotics (25).

Earlier studies have documented that the SOS response is an extremely important molecular instrument of bacteria which allows it to deal with diverse DNA damages (26), but at the same time, activation of the SOS response accelerates the synthesis of the SulA protein, which can arrest cell division (27). In the present study, strain EΔ*hns* not only grew slowly, but also showed cell membrane damage. Therefore, we speculate that the deletion *hns* gene in *E. coli* ATCC 25922 leads to bacterial membrane damage, thus activating the SOS response, which in turn hinders bacterial growth.

### 3.7 Increased proton motive force facilitates the uptake of aminoglycosides

It is well-known that aminoglycosides do not require energy to cross the OM of bacteria, where OM damage and increased expression of porin-related genes can accelerate absorption, while PMF is required to provide energy when they pass through the IM (28–30). Bacterial PMF, an energetic pathway located on the bacterial membrane, consists of Δψ and the transmembrane proton gradient (ΔpH).

To verify whether PMF altered among strains *E. coli* ATCC 25922, EΔ*hns* and EΔ*hns*/p*hns*, we first assessed the dissipation of Δψ by the fluorescent probe 3,3′-Dipropylthiadicarbocyanine Iodide [DiSC_3_ (5)]. When Δψ dissipates, the fluorescent probe DiSC_3_ (5) is released into the buffer solution, resulting in a significant increase in fluorescence intensity. Our findings showed that compared with *E. coli* ATCC 25922, the fluorescence intensity of strain EΔ*hns* was decreased dramatically (*P* < 0.0001) (Figure 4C), indicating that the dissipation of Δψ in strain EΔ*hns* was sharply repressed. Further, the fluorescence intensity of complemented strain EΔ*hns*/p*hns* reverted to the levels of *E. coli* ATCC 25922. Similarly, ΔpH was also determined by the fluorescent probe BCECF-AM (20 μM, Beyotime, Shanghai, China) and the results demonstrated that no observable changes were found in the tested bacteria (Figure 4D). These results imply that the elevated PMF of strain EΔ*hns* is mainly due to the inhibition of Δψ dissipations, which enhances the uptake of aminoglycosides.

### 3.8 Lower efflux pumps activity reduces drugs efflux of strain EΔ*hns*

Given that the intracellular accumulation of antibiotics is determined by both influx and efflux (31), we also determined efflux pumps activity changes in *E. coli* ATCC 25922, EΔ*hns* and EΔ*hns*/p*hns* using ethidium bromide as a hydrophobic fluorescent probe (Figure 4E). Compared with *E. coli* ATCC 25922 and complementary strain EΔ*hns*/p*hns*, the fluorescence intensity of strain EΔ*hns* exhibited higher (*P* < 0.0001), suggesting that its efflux activity was significantly inhibited.

### 3.9 Activation of glycolysis and electron transport chain leads to an increase of PMF in strain EΔ*hns*

**Figure 6:**
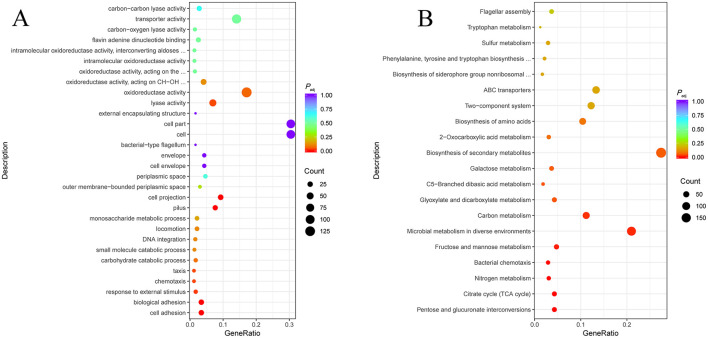
Transcriptomic analysis of strain EΔ*hns* against the reference genome of *E. coli* ATCC 25922. **(A)** Gene Ontology (GO) function classification of differentially expressed genes (DEGs). **(B)** Kyoto Encyclopedia of Genes and Genomes (KEGG) pathway enrichment analysis of DEGs. *P*_adj_ < 0.05 is statistically significant. The x axis and y axis indicate the name of the pathway and the Gene ratio, respectively. The Gene ratio is the ratio of the number of differentially expressed genes enriched into a particular pathway or functional class to the number of all annotated genes in that pathway or functional class. The size of the dot indicates the number of differentially expressed genes in this pathway. The colors of the points correspond to different *P*_adj_ ranges. The strain EΔ*hns* is the derivative of *E. coli* ATCC 25922 that lacks *hns* and is induced by 0.2% L-arabinose.

To gain a deeper understanding of H-NS regulation mechanism on the susceptibility of *E. coli*, we further performed the transcriptomic analysis. In comparison with the control *E. coli* ATCC 25922, Gene Ontology (GO) annotation analysis (Figure 6A) showed that DGEs of strain EΔ*hns* were mainly correlated with adhesion, stress response and chemotaxis of biological processes, lyase activity of molecular function, and pili of cellular component. Kyoto Encyclopedia of Genes and Genomes (KEGG) enrichment analysis (Figure 6B) revealed that DEGs in strain EΔ*hns* were enriched significantly in energy metabolisms.

**Figure 7:**
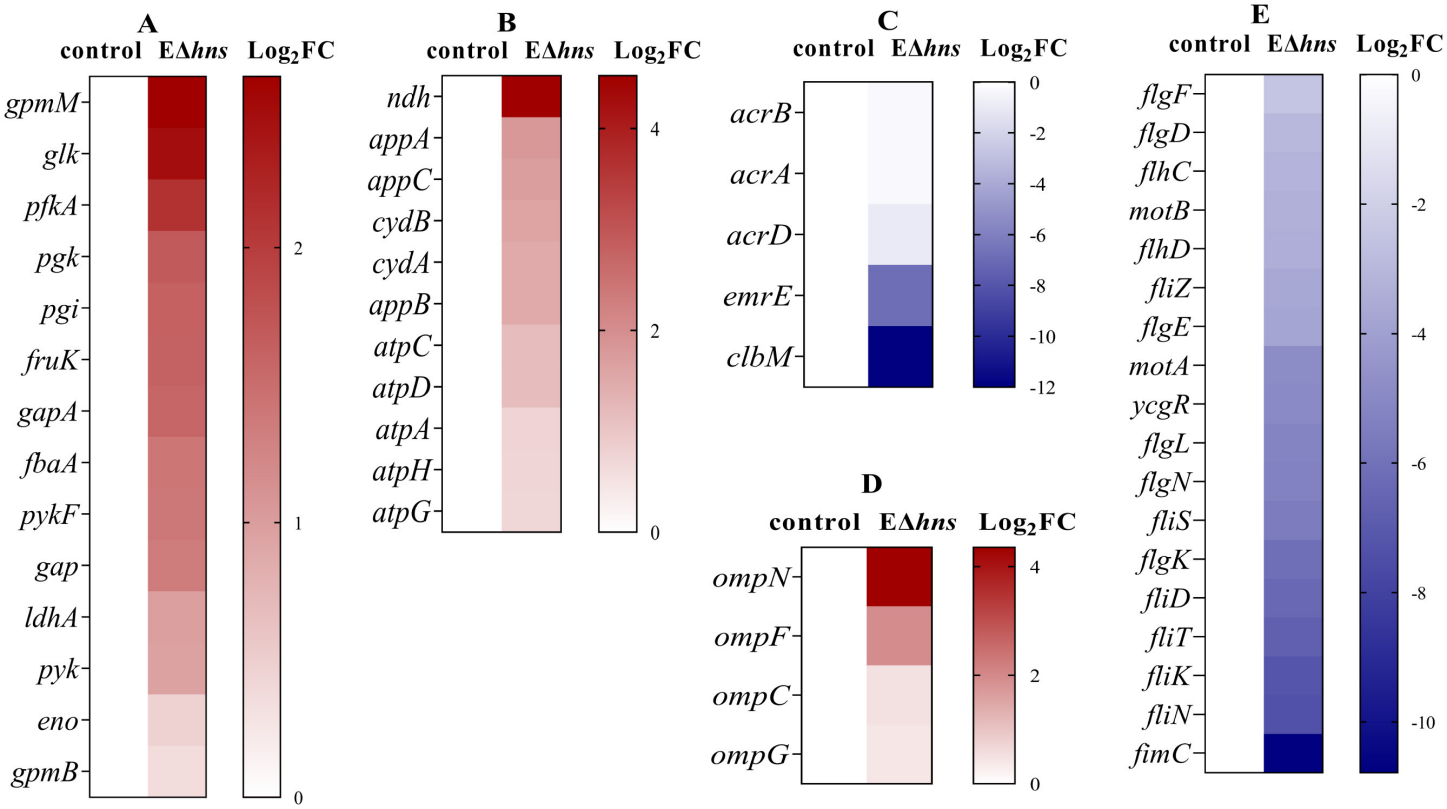
Differentially expressed genes (DEGs) obtained from *E. coli* ATCC 25922 and EΔ*hns*. *E. coli* ATCC 25922 is the control. The strain EΔ*hns* is the derivative of *E. coli* ATCC 25922 that lacks *hns*. **(A)** DEGs involved in the glycolysis. **(B)** DEGs involved in the electron transport chain. **(C)** DEGs involved in efflux systems. **(D)** DEGs involved in porins. **(E)** DEGs involved in flagellar systems. Log_2_FC means log_2_ (Fold Change), blue for decreased expression, brown for increased expression. The strain EΔ*hns* is the derivative of *E. coli* ATCC 25922 that lacks *hns* and is induced by 0.2% L-arabinose.

Specifically, the genes with significantly increased expression were involved in glycolysis and electron transport chain of respiratory process (Figure 6) in strain EΔ*hns*. As shown in Figure 7A, the expression levels of glycolytic-related genes presented distinctly elevated. In parallel, the genes involved in respiratory chain, such as NADH-Q oxidoreductase-related gene *ndh* and cytochrome oxidase-related genes *appA, appB, appC, cydA*, and *cydB*, were all obviously up-regulated (Figure 7B). Previous studies have verified that an increase in glycolytic metabolites can facilitate the conversion of redox energy to PMF, thereby promoting aminoglycosides uptake (28, 32, 33). Accordingly, to identify whether the respiratory chain is activated, we determined the intracellular NAD^+^/NADH ratio in strains EΔ*hns*, EΔ*hns*/p*hns* and *E. coli* ATCC 25922 using an NAD^+^/NADH Assay Kit (WST-8, Beyotime, Shanghai, China). As expected, the NAD^+^/NADH ratio of strain EΔ*hns* was remarkably higher than that of *E. coli* ATCC 25922 (*P* < 0.0001) (Figure 4F), suggesting that the deletion *hns* gene in *E. coli* ATCC 25922 can stimulate glycolysis and promote the conversion of redox energy into PMF, thus facilitating the uptake of aminoglycosides.

**Figure 8:**
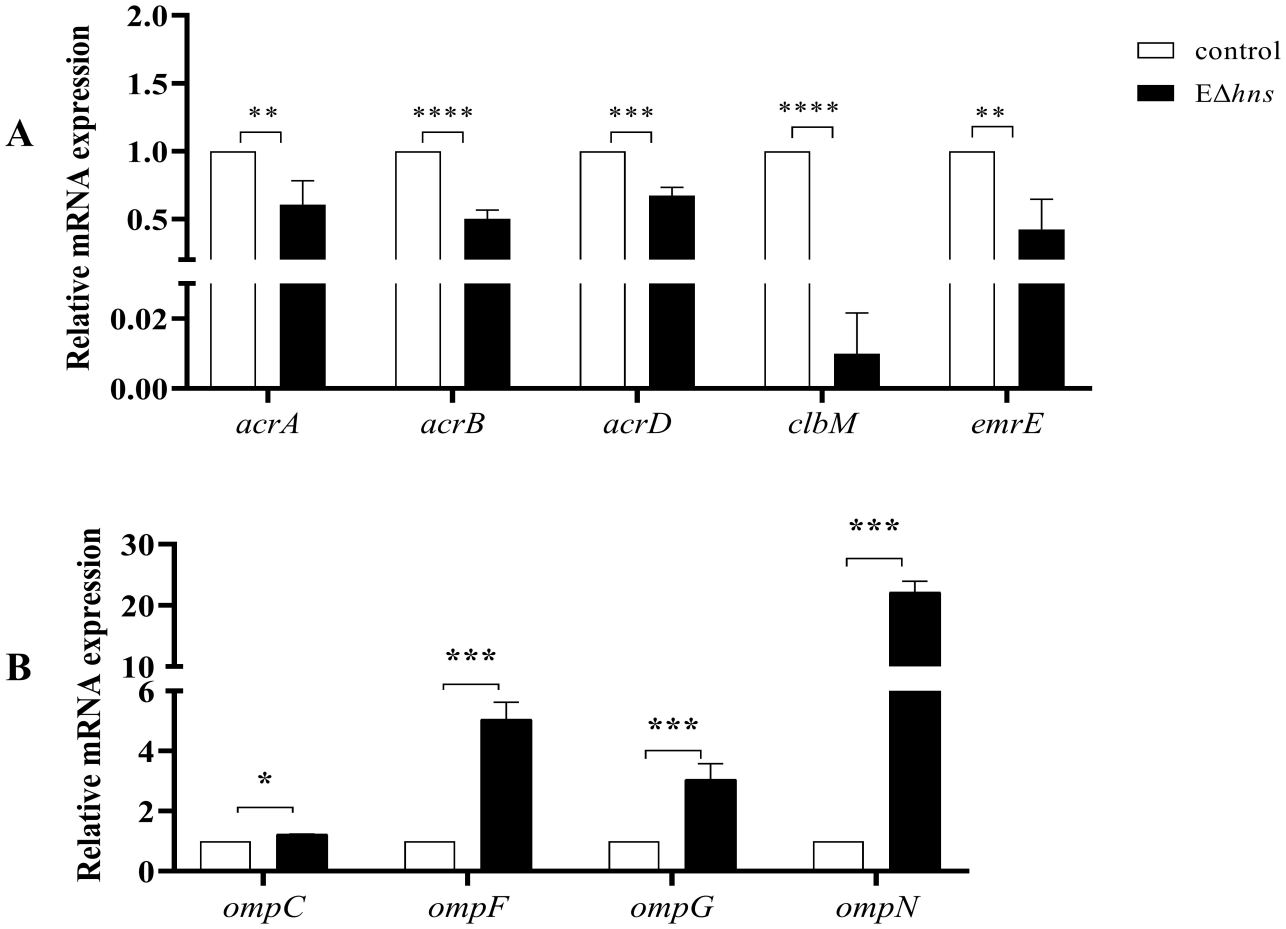
The mRNA expression levels of strains *E. coli* ATCC 25922, EΔ*hns* and EΔ*hns*/p*hns*. **(A)** Efflux-related genes. **(B)** Porin-related genes. *E. coli* ATCC 25922 is the control. The strain EΔ*hns* is the derivative of *E. coli* ATCC 25922 that lacks *hns* and is induced by 0.2 % L-arabinose. ns, No significant difference by Student's *t*-test. *; *P* < 0.05, **; *P* < 0.01; ***, *P* < 0.001; ****, *P* < 0.0001.

Further analysis found that many efflux-related genes (Figure 7C) presented down-regulated, such as *clbM* (34), *emrE* (35, 36), *acrD* (37), and *acrAB* (38), which were helpful to reduce drugs efflux. To confirm whether the expression levels of above genes were altered in strain EΔ*hns*, we examined their mRNA relative expression levels using RT-qPCR (Figure 8A). The results demonstrated that the expression levels in strain EΔ*hns* were evidently lower than those in *E. coli* ATCC 25922, approximately decreased by 99.0% (*clbM*), 68.3% (*emrE*), 49.7% (*acrB*), and 39.2% (*acrA*), respectively. Thus, reduced expression levels of genes associated with the efflux system can inhibit efflux activity, thereby helping to slow drugs efflux.

Due to porin-related genes *ompN, ompF, ompC*, and *ompG* (Figure 7D) showed obvious upregulations, we also examined their mRNA expression levels in strains *E. coli* ATCC 25922 and EΔ*hns* using RT-qPCR. We found that the expression levels of these genes were markedly higher in strain EΔ*hns* than that in *E. coli* ATCC 25922 (Figure 8B), with the highest levels of *ompN* (i.e., 22.21-fold higher), followed by *ompF* (5.06-fold higher) and *ompG* (3.06-fold higher), whereas the levels of *ompC* exhibited only a marginal increase. Porins OmpC and OmpF passively diffuse small hydrophilic molecules (≤ 500 Da) into the periplasm (39), and their expressions are mutually exclusive, i.e., when ompF is on, ompC is off, and vice versa (40). OmpG, which belongs to the subclass of porins, harbors a larger channel than OmpF and OmpC, and also allows for translocation (41). OmpN is one of the minor porins, although its translocation function has not been reported (42). Therefore, it is conceivable that the elevated expression levels of porin-related genes will further destabilize the OM barrier and contribute to the increase of OM permeability of strain EΔ*hns*.

Intriguingly, there were many down-regulated DEGs in the flagellar system in strain EΔ*hns* (Figure 7E), compared to the reference strain *E. coli* ATCC 25922. Further, it can also be seen from Figure 5 that the flagella of *E. coli* ATCC 25922 are clearly visible, while that of strain EΔ*hns* is almost invisible. Many studies have revealed that flagellum is a locomotive organelle and can affect bacterial adhesion, invasion and biofilm formation (37, 43). To further confirm whether the motility of strain EΔ*hns* has changed, we fulfilled the swimming motility assay of strains *E. coli* ATCC 25922, EΔ*hns*, EΔ*hns*/p*hns*, and E/p*hns* using LB plates with 0.3% agar according to the method of previous studies (44, 45). The results demonstrated that strain EΔ*hns* had the shortest swimming distance (≈ 9.3 mm), followed by EΔ*hns*/p*hns* (≈ 12.8 mm) and *E. coli* ATCC 25922 (≈ 13.8 mm), while E/p*hns* had the longest distance (≈ 14.7 mm), suggesting that H-NS modulates the motility of *E. coli* by positively governing the expression of flagellate-related genes.

## 4 Conclusion

**Figure 9:**
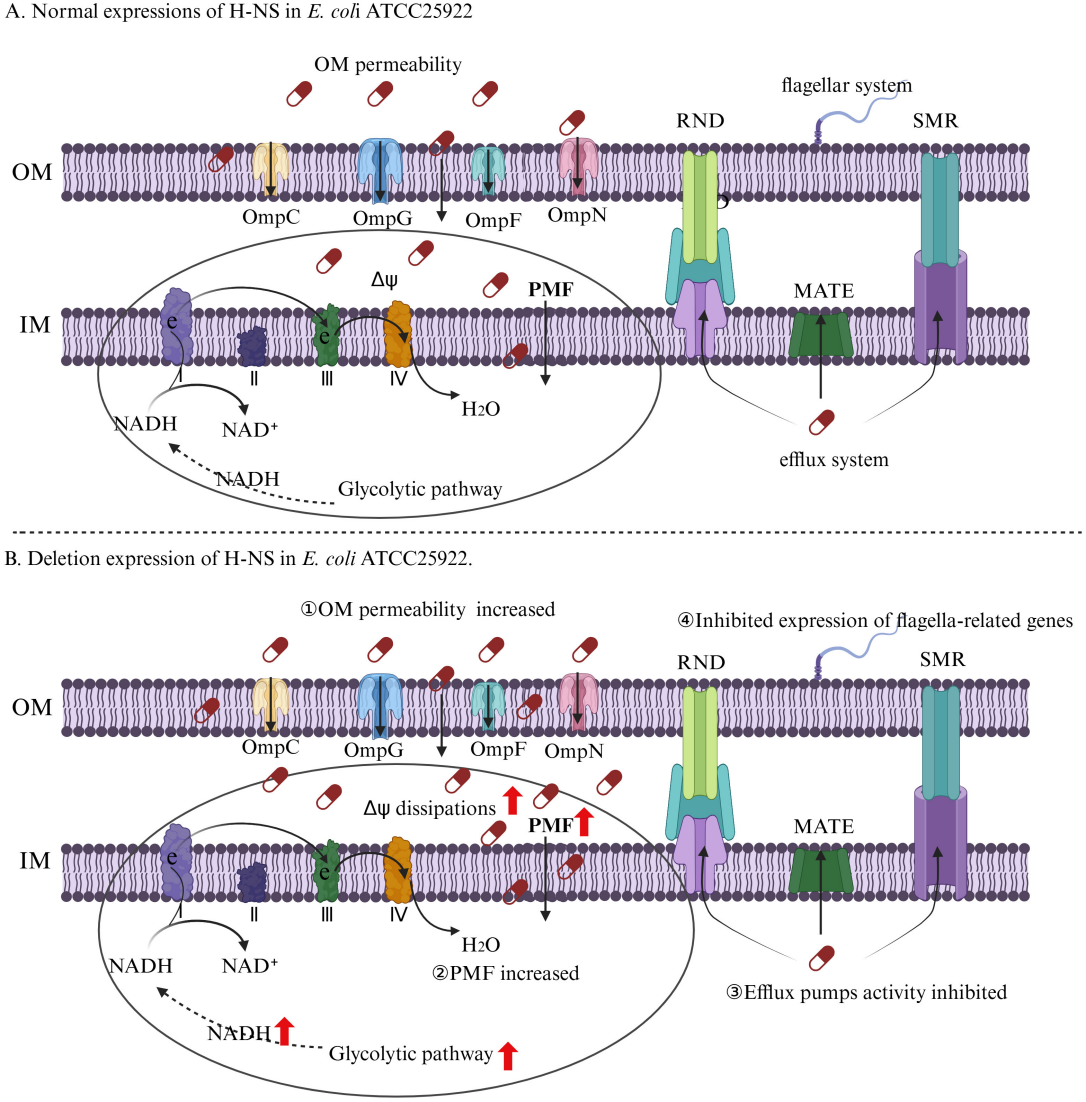
The regulation mechanism of H-NS on aminoglycosides resistance of *E. coli*. **(A)** *E. coli* ATCC 25922. **(B)** *E. coli* ATCC 25922 that lacks *hns*. I, II, III, and IV represent complexes I, complexes II, complexes III, and complexes IV, which are respiratory chain complex and carries out electron transport. The OM permeability of strain EΔ*hns* is improved by membrane damages and increased expression levels of porin-related genes, thus increasing the uptake of aminoglycosides. The activation of glycolysis and electron transport chain and the inhibition of Δψ dissipations in strain EΔ*hns* result in an increase of PMF, which can further facilitate the uptake of aminoglycosides. The efflux pumps activity of strain EΔ*hns* is inhibited that can reduce drugs efflux. The flagella of strain EΔ*hns* is apparently reduced, which can inhibit the formation of bacterial biofilms, thereby enhancing bacterial susceptibility to aminoglycosides.

Taken together, our findings highlight the deletion of *hns* in *E. coli* can strengthen antibacterial activity of aminoglycosides by increasing intracellular drug concentrations (Figure 9). On the one hand, the inhibition of efflux pump activity in strain EΔ*hns* can reduce the efflux of aminoglycosides. On the other hand, in addition to elevated OM permeability can promote drugs uptake, the increase of PMF induced by glycolysis and Δψ can further accelerate the uptake of aminoglycosides.

## Data Availability

The datasets presented in this study can be found in online repositories. The names of the repository/repositories and accession number(s) can be found in the article/supplementary material.
